# Supplementary material for: Evolutionarily informed machine learning enhances the power of predictive gene-to-phenotype relationships
Source: Nat Commun. 2021 Sep 24;12:5627. doi: 10.1038/s41467-021-25893-w (PMC8463701; doi:10.1038/s41467-021-25893-w)
Supplement: Supplementary file 1 — Supplementary Information [file 41467_2021_25893_MOESM1_ESM.pdf]

Evolutionarily informed machine learning enhances the power of predictive gene-to-phenotype relationships

Chia-Yi Cheng[1,2], Ying Li[3], Kranthi Varala[3], Jessica Bubert[4], Ji Huang[1], Grace J Kim[1], Justin Halim[1], Jennifer Arp[4], Hung-Jui S Shih[1], Grace Levinson[1], Seo Hyun Park[1], Ha Young Cho[1], Stephen P Moose[4], Gloria M Coruzzi[1]

[1]Center for Genomics and Systems Biology, Department of Biology, New York University, New York, NY 10003, USA;

[2]Current Affiliation: Department of Life Science, National Taiwan University, Taipei, Taiwan;

[3]Department of Horticulture and Landscape Architecture, Purdue University, West Lafayette, IN, USA; Purdue Center for Plant Biology, Purdue University, West Lafayette, IN, USA

[4]Department of Crop Sciences, University of Illinois at Urbana-Champaign, Urbana, IL 61801

Correspondence and requests for materials should be addressed to G.M.C.
(email:gloria.coruzzi@nyu.edu)

Figure S1

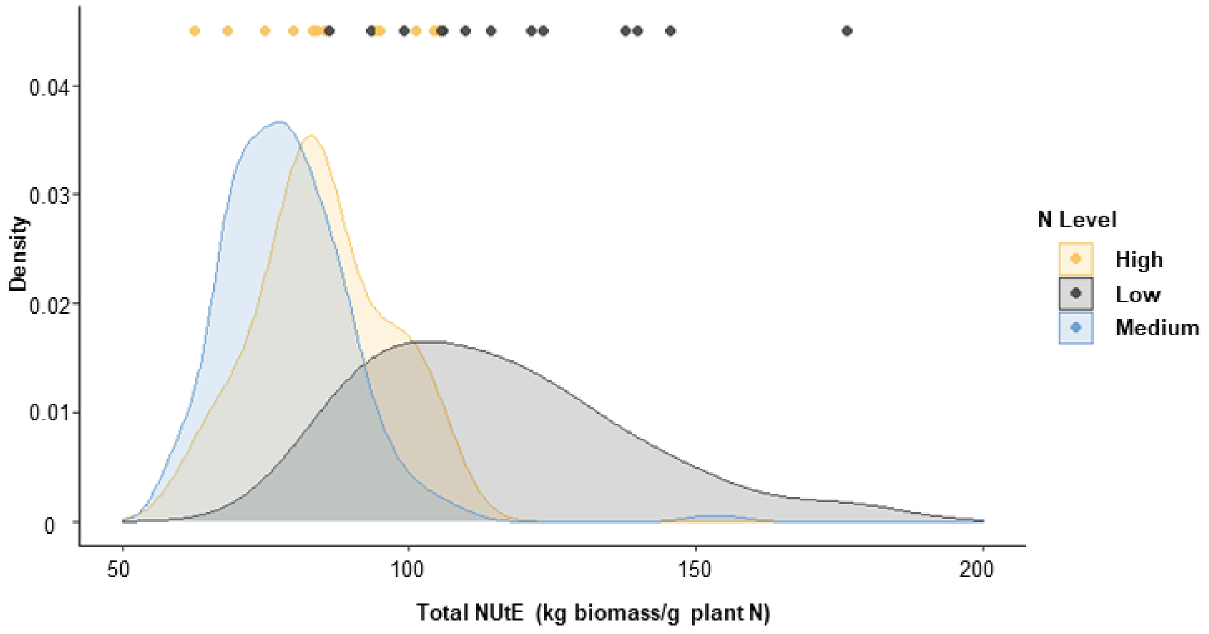

Supplementary Figure 1. Distribution of nitrogen utilization values among U.S. Corn Belt inbred diversity and the genotypes chosen for transcriptome-based prediction of this trait. Total NUtE in a core set of 25 inbreds grown with excess N (High, yellow) overlaps with the distribution of the larger population of >300 genotypes grown with sufficient N (Medium, blue). Total NUtE increases for the core 25 inbreds grown with limiting soil N supply (Low, grey). The total NUtE values for the 12 inbreds chosen for transcriptome-based prediction of the trait are shown as yellow (High) or grey (Low) circles, which again span the range of values in the entire population. The source data for this figure is provided in Supplementary Table 1.

Fig. S2

a

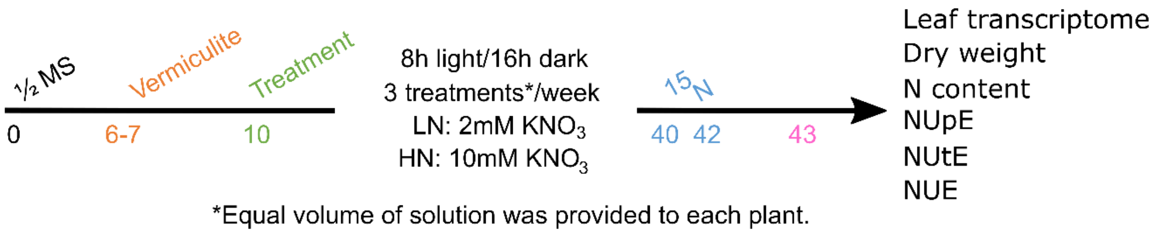

b

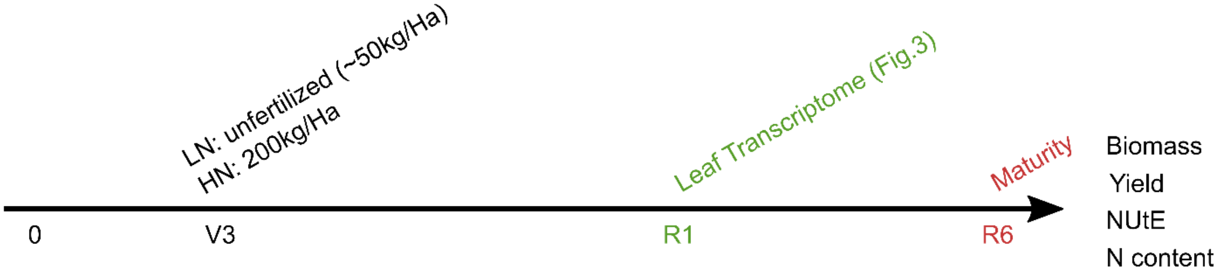

Supplementary Figure 2. Schematic overview of plant growth conditions and N-treatments. (a) Eighteen accessions of Arabidopsis grown in the nutrient poor matrix vermiculite were supplemented with trackable amount of potassium nitrate (Methods). The RNA samples and phenotypic measurements were collected at the vegetative stage right before bolting. (b) A collection of maize inbreds and their hybrids with B73 were grown in the field in Urbana, Illinois that have been shown to be responsive to N fertilizer. The leaf RNA samples were collected at anthesis (R1 stage) and the phenotypes were scored at the physiological maturity (R6 stage).

Figure S3

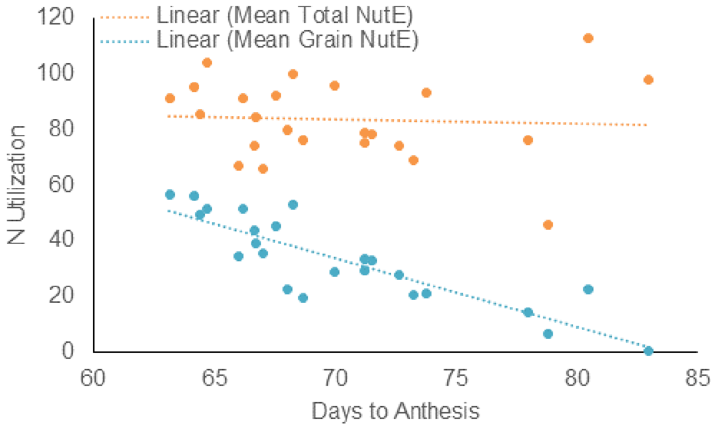

Supplementary Figure 3. In maize, total NUtE is an optimal measure of NUE, compared to grain NUtE, the latter of which is confounded by maturity. The maize genotypes used in this study with diverse days to anthesis were plotted for grain and total NUtE. The regression lines show that grain NUtE is confounded by maturity, while total NUtE is not.

Figure S4

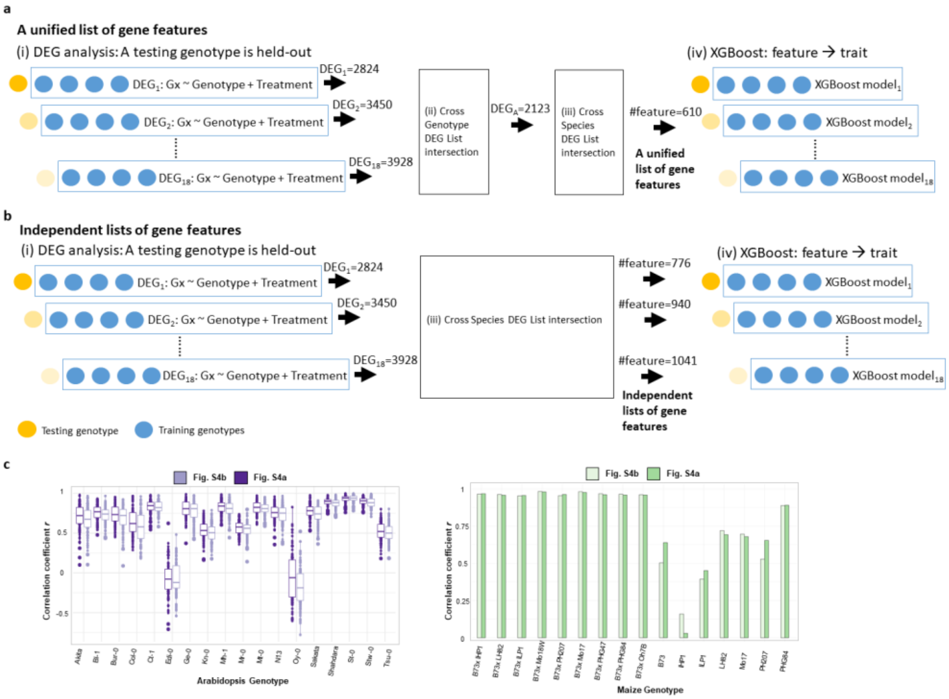

Supplementary Figure 4. Comparison of XGBoost models created using a unified list of gene features (a), or independent lists of gene features (b). **(a)** Unified list of gene features: For each round of DEG analysis, a test genotype was held-out and not included in the DEG analysis. This resulted in 18 independent DEG lists for Arabidopsis (a, i). These 18 independent DEG lists were then intersected to generate a unified list of DEGs within species (a, ii). Next, the DEGs shared by all 18 Arabidopsis genotypes were used for cross-species intersection with maize (a, iii). This approach resulted in a unified list of gene features which was used in XGBoost models (a, iv). **(b)** Independent list of gene features: This approach used 18 independent DEG lists within Arabidopsis (b, i), and each list was intersected independently in a cross-species analysis with maize (b, iii), and used in XGBoost models in (b, iv). (Note: The numbers of gene features shown in Panels a and b are for Arabidopsis. However, the 16 maize samples were processed using this same pipeline). **(c)** Model performance: The XGBoost performance using a unified list of DEGs (a), is comparable to the independent DEG list (b), for both Arabidopsis and maize. The advantage of using the unified list approach (a), as done in this study, is that it generates a ranked list of gene feature importance that is relevant across many genotypes. In Arabidopsis, the boxplot shows the Pearson's *r* of 100 iterations. The box represents the 25th to 75th percentile and the line within the box marks the median. Whiskers above and below the box indicate the 10th and 90th percentiles. Points above and below the whisers indicate outliers outside 10th and 90th percentiles. In maize, there are only two data points for each genotype thus the Pearson's *r* was calculated from the pooled predicted and actual NUE from 100 iteration.

Figure S5

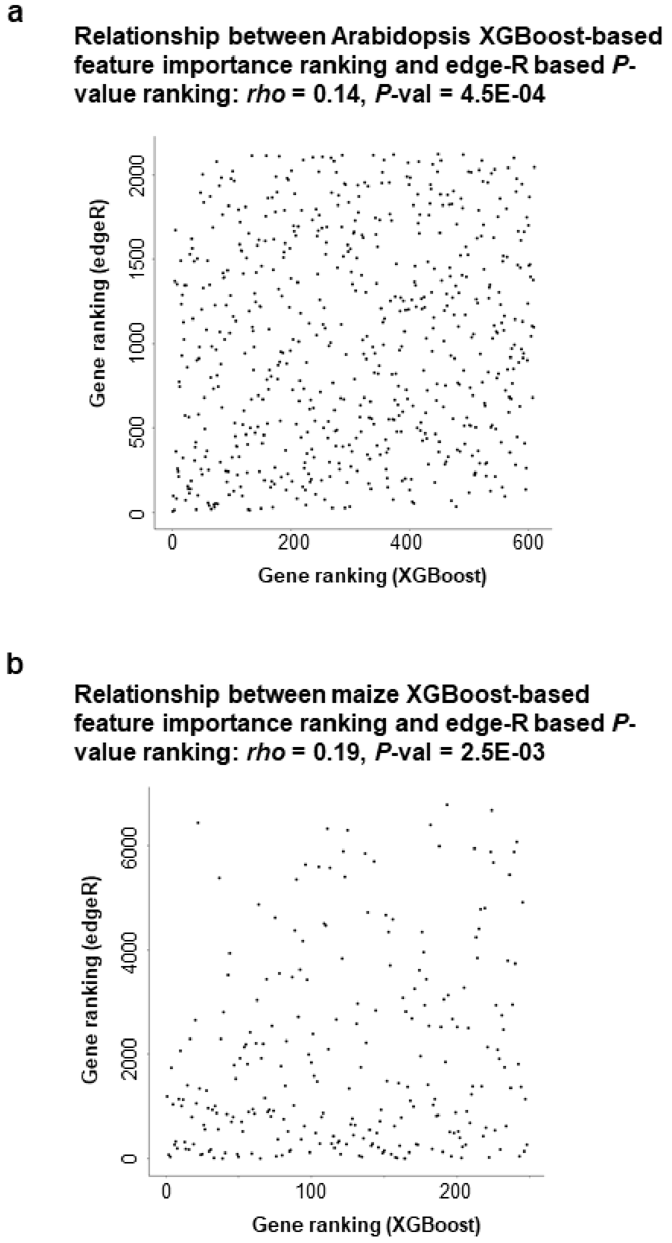

Supplementary Figure 5. XGBoost-based feature importance ranking is marginally correlated with the edgeR-based *P*-value ranking. The scatterplots show the relationship between the XGBoost-based gene feature importance and the edgeR-based *P*-value ranking in Arabidopsis (a) and maize (b). The marginal correlation coefficients suggest that the gene-trait relationship revealed by XGBoost was more than simple N response: the XGBoost-based gene feature importance revealed non-linear relationship between transcript abundance and the trait. The *P*-val was calculated using the default option for cor.test (method = "spearman") in R.

Figure S6

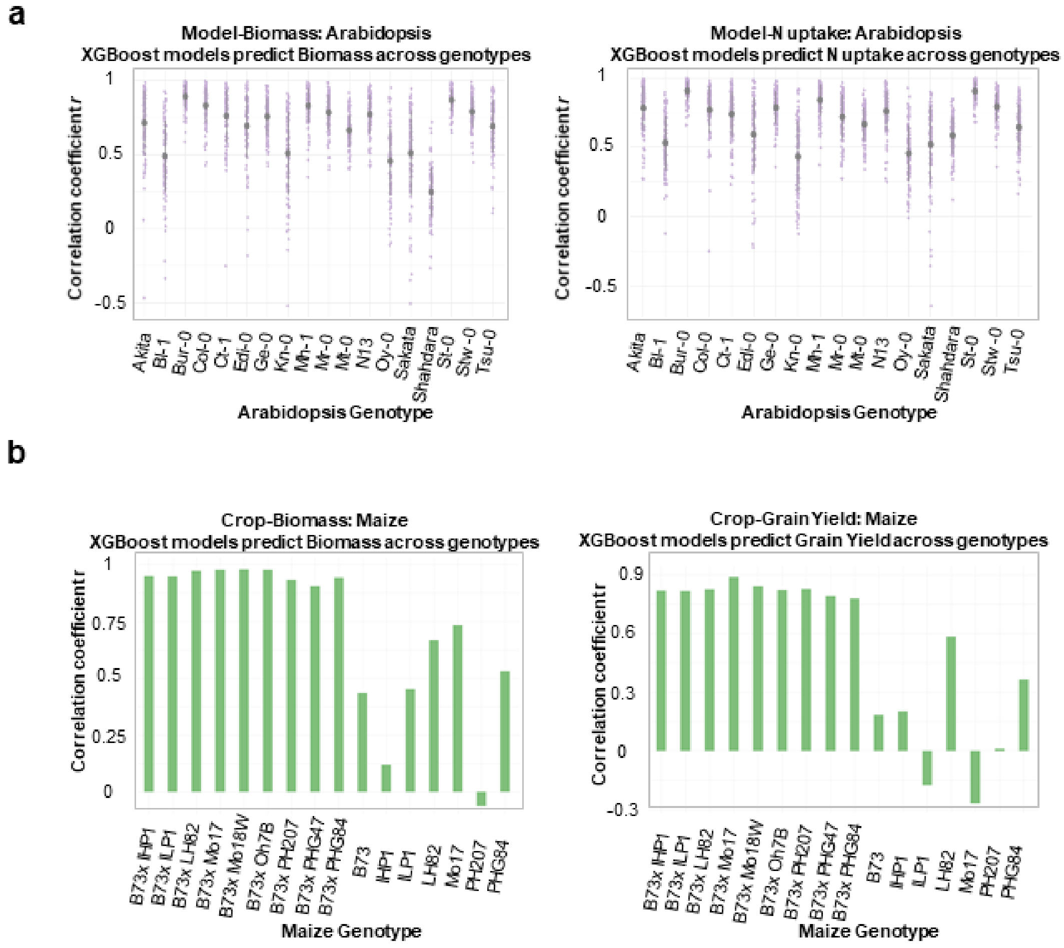

Supplementary Figure 6. The conserved N-DEGs can be used to predict multiple traits. We used the conserved N-DEGs to predict additional traits in Arabidopsis (a) and maize (b). The model performance was evaluated by calculating the Pearson's correlation coefficient *r* between the predicted and actual NUE values. In Arabidopsis, the dots indicate the Pearson's *r* of 100 individual iterations and the pointranges indicate mean +/- SD. In maize, there are only two data points for each genotype thus the Pearson's *r* was calculated from the pooled predicted and actual NUE from 100 iteration. For all four species-trait combinations, the XGBoost models using the conserved N-DEGs as features outperformed those using the same number of random expressed genes. The complete XGBoost gene feature ranking is provided in Supplementary Table 3.

Figure S7

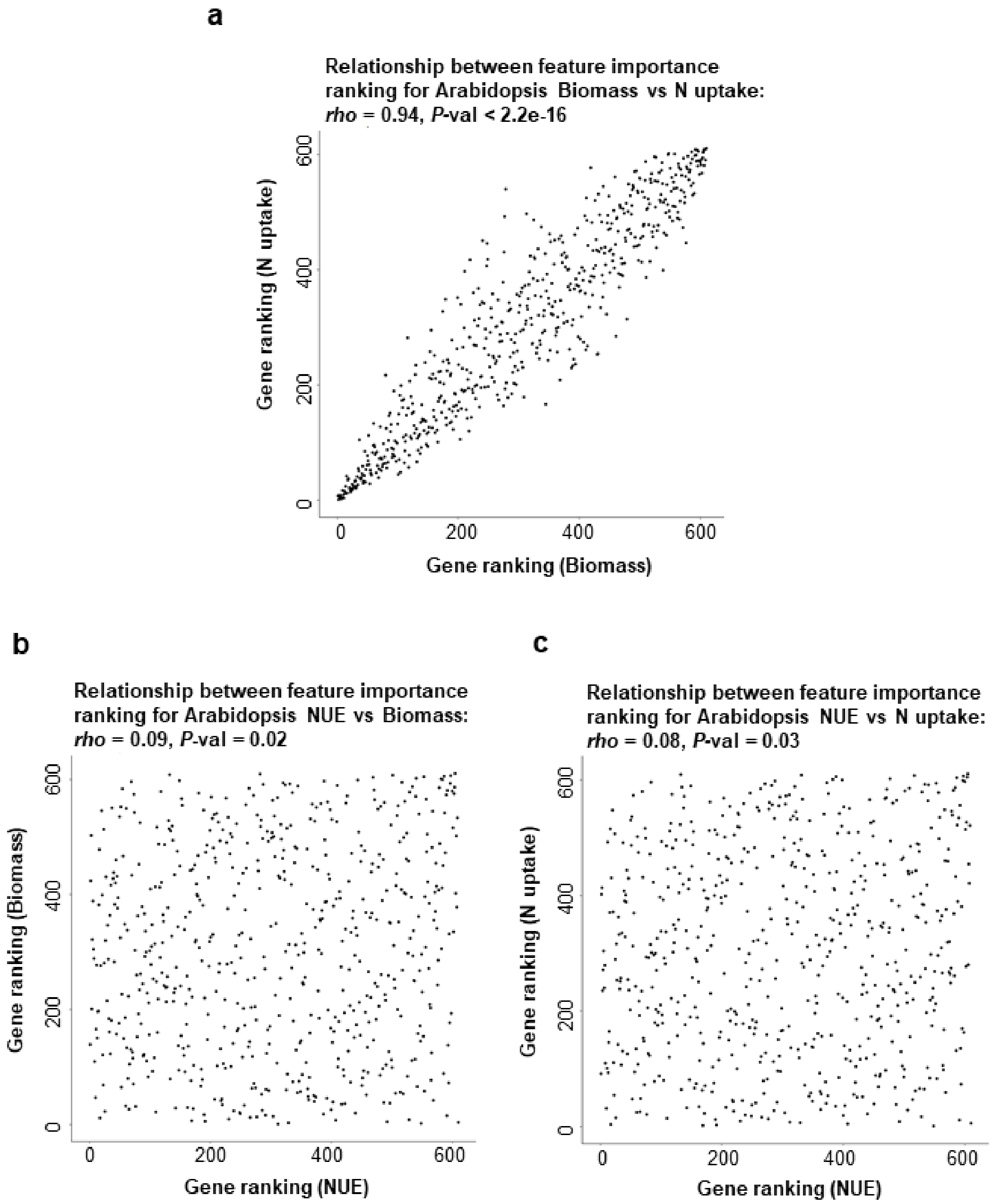

Supplementary Figure 7. The Arabidopsis gene feature importance ranking is trait specific. The scatterplots show the relationship between the XGBoost-based gene feature importance ranking among different models. (a) The ranking in Biomass models is highly correlated (*rho* = 0.94) with that in N uptake model, which can be explained by the correlation between biomass and N uptake (*r* = 0.97, Fig. 2b). Similarly, the ranking in NUE model is marginally correlated with the rankings in Biomass (b) and N uptake (c) models, which is consistent with the low correlation between traits ($r_{\text{NUE and Biomass}}$ = 0.14, $r_{\text{NUE and N uptake}}$ = 0.01, Fig 2b). The source data are provided in Supplementary Tables 2 and 3. The *P*-val was calculated using the default option for cor.test (method = "spearman") in R.

Figure S8

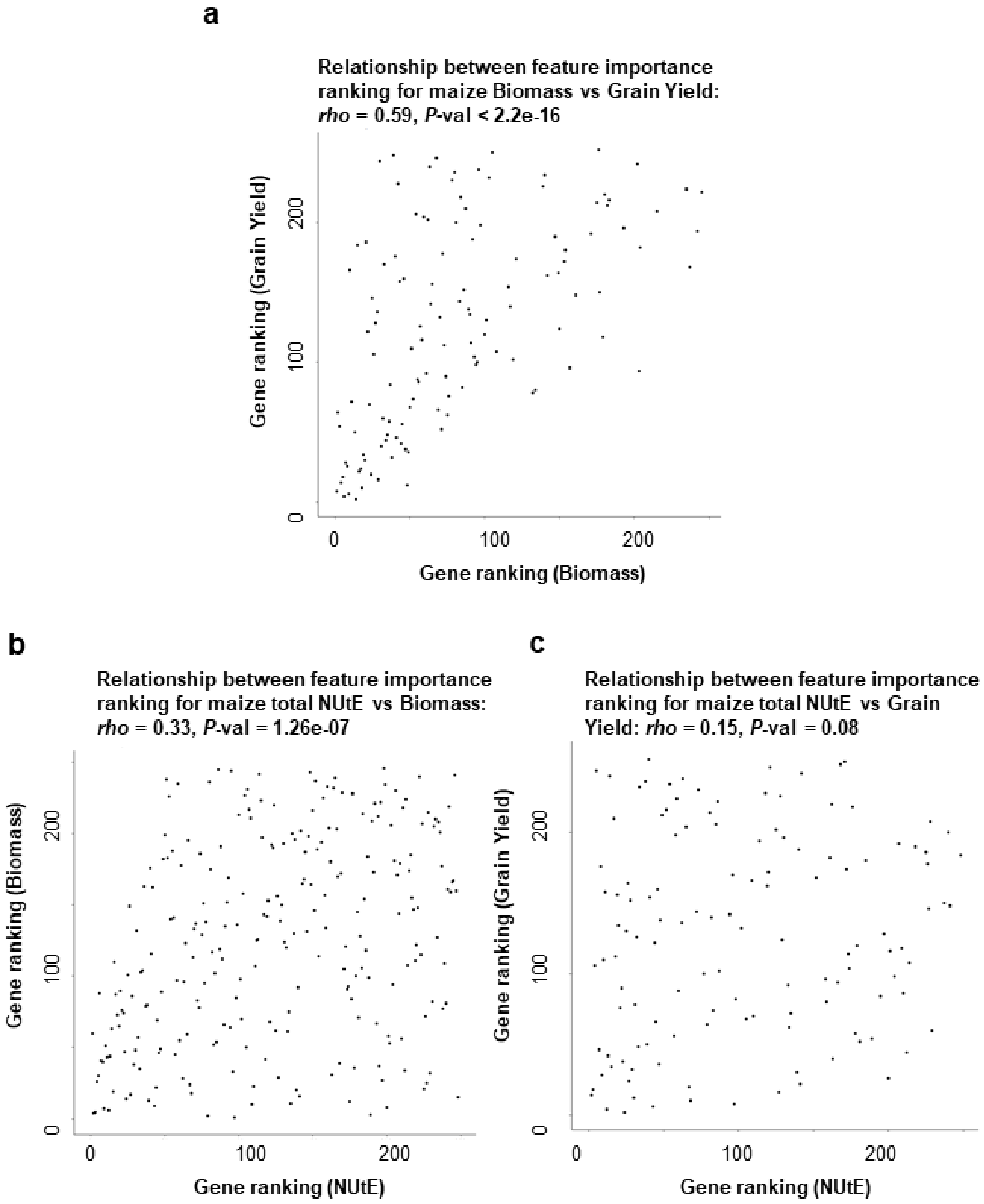

Supplementary Figure 8. The maize gene feature importance ranking is trait specific. The scatterplots show the relationship between the XGBoost-based gene feature importance ranking from different models. (a) The ranking in Total Biomass models is positively correlated (*rho* = 0.59) with that in Grain Yield model, which can be explained by the correlation between biomass and N uptake (r = 0.8, Fig. 3b). Similarly, the ranking in Total NUtE model is marginally correlated with the rankings in Total Biomass (b) and Grain Yield (c) models, which is consistent with the low correlation between traits ($r_{\text{NUtE and Biomass}}$ = -0.14, $r_{\text{NUtE and Grain Yield}}$ = -0.19, Fig 3b). The source data are provided in Supplementary Tables 2 and 3. The *P*-val was calculated using the default option for cor.test (method = "spearman") in R

Figure S9

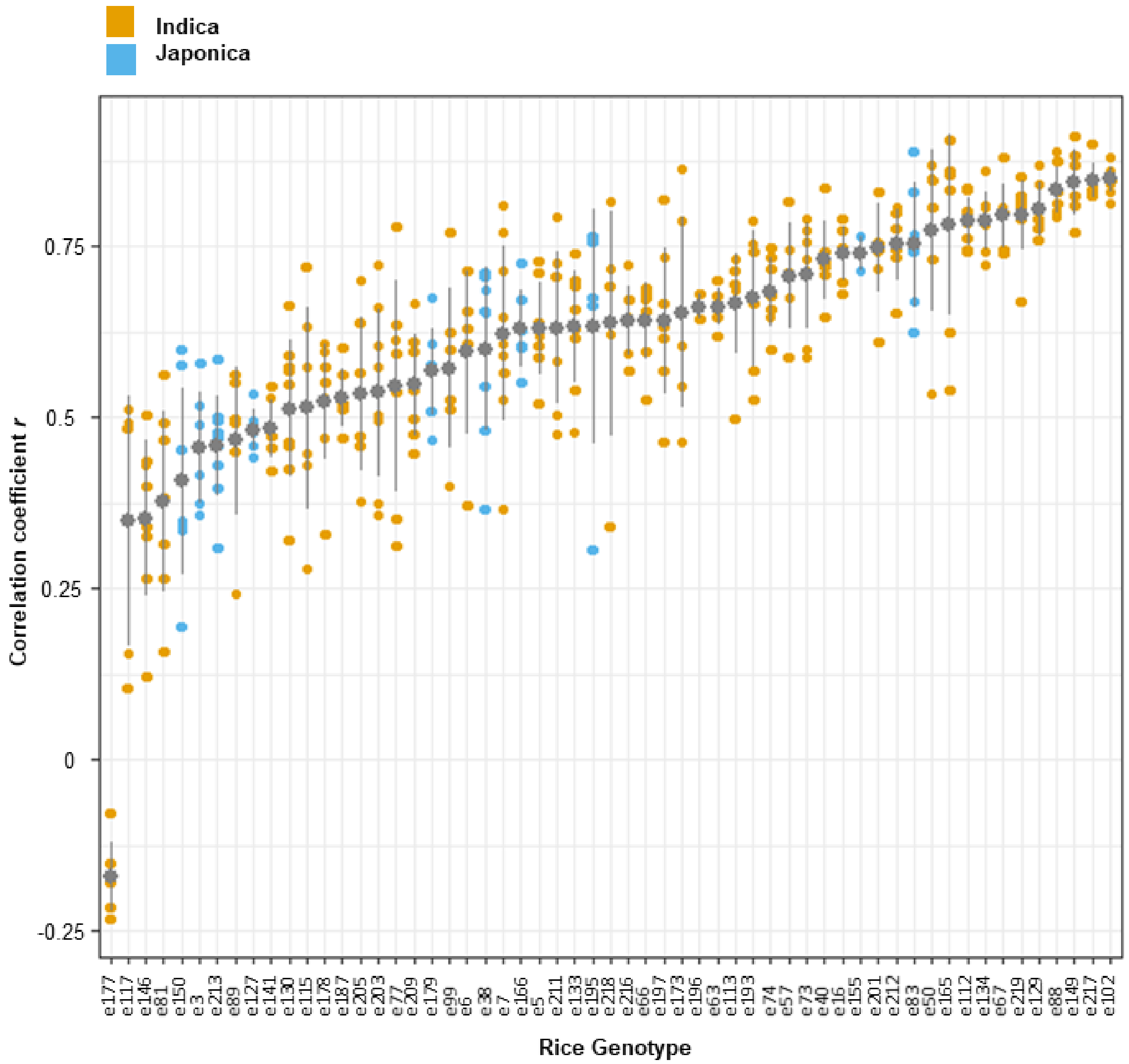

Supplementary Figure 9. Use case: the pipeline proposed in this study can be applied on a different data set. We used the pipeline on a rice data set (Groen *et al.,* 2020) consisting of transcriptomic and phenotypic data from 220 genotypes subjected to drought treatment. We identified drought-DEGs from 20 randomly selected genotypes as gene features for predicting the fecundity in the left-out 37 genotypes. We repeated the random selection 10 times and the mean Person's *r* was 0.62. The performance was consistent across japonica and indica genotypes. For each of the 10 random selection, the XGBoost models using drought-DEGs outperformed the ones using the same number of random expressed genes (one-tailed Mann–Whitney U test, *P*-value < 2.2e-16). The pointranges indicate mean +/- SD. The mapping file for the genotype identifiers (e#) and the full name is provided in Supplementary Table 9.

Figure S10

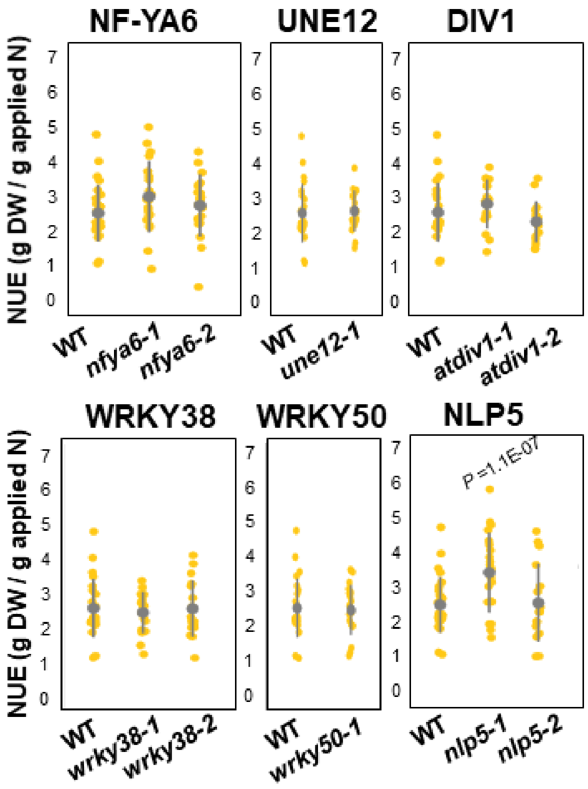

Supplementary Figure 10. Validation of candidate TFs in NUE using loss-of-function mutants in Arabidopsis. The NUE of the Arabidopsis mutants in Group II genes were similar to that in the wildtype (WT). This indicates the Group II genes are either not required or functionally redundant under N-replete condition. The experiments were caried out three times with 10 or more plants per genotype per condition. The pointrange indicates mean +/- SD. The *P*-value was calculated between WT and indicated mutant allele using one-sided t-test with unequal variance.

Figure S11

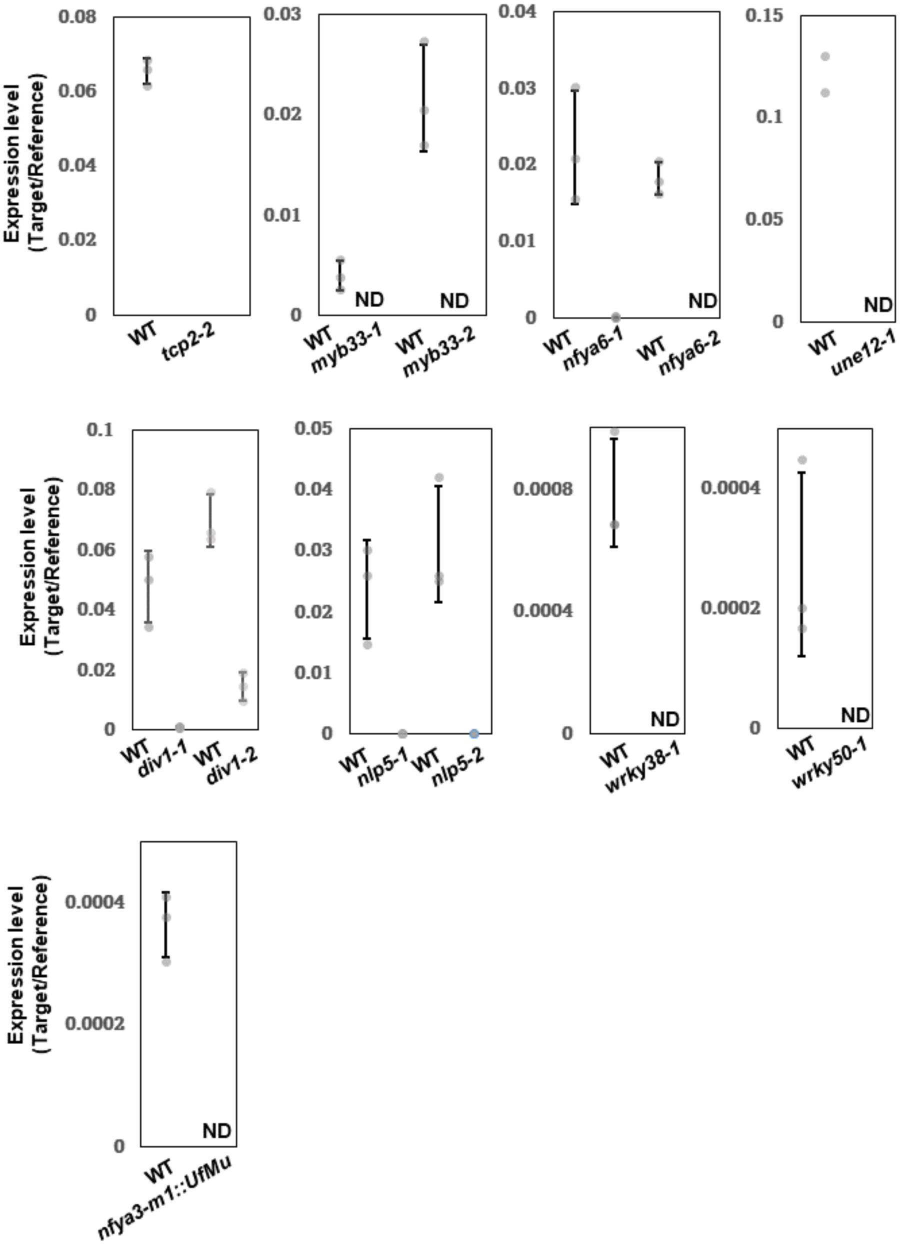

Supplementary Figure 11. Expression of target genes in plant loss-of-function mutants used in this study. To confirm the knockout of full-length transcript expression in the Arabidopsis or maize mutants, we designed primers spanning the insertion site for each allele. For mutants, each RNA sample was isolated from a pool of three or more plants whose homozygosity were confirmed using genotyping. The dots are calculated from technical replicates and the pointranges indicate mean +/- SD. Only the mutants with reduced or knockout expression levels (Target/Reference compared to wildtype) were retained for functional validation analysis. WT: wildtype, ND: Not detected, CT > 45. Reference: TUB4 for Arabidopsis and UBQ for maize. The primer information is provided in Supplementary Table 6.
