# Supplementary material for: Evolutionarily informed machine learning enhances the power of predictive gene-to-phenotype relationships
Source: Nat Commun. 2021 Sep 24;12:5627. doi: 10.1038/s41467-021-25893-w (PMC8463701; doi:10.1038/s41467-021-25893-w)
Supplement: Supplementary file 3 — Description of Additional Supplementary Files [file 41467_2021_25893_MOESM3_ESM.pdf]

Description of Additional Supplementary Files

File Name: Supplementary Data 1
Description: A summary of the maize physiological traits for >300 genotypes in 2014. This is the input table for Figure S2.

File Name: Supplementary Data 2
Description: Lists of differentially expressed genes in Arabidopsis and maize. For each species, the first round of DEG analysis (FDR<0.05) was conducted using n-1 genotypes (n=number of genotypes) (See Fig S4 for details). Each sub-table represents the intersected DEGs, which means they appeared in n DEG lists during the first round of DEG analysis. Theses DEGs were ranked based on the mean P-value in the first round of DEG analysis.

File Name: Supplementary Data 3
Description: Ranking of Arabidopsis genes based on the sum of feature importance score from eighteen XGBoost biomass models.

File Name: Supplementary Data 4
Description: The Arabidopsis TFs ranked based on the number of edges.

File Name: Supplementary Data 5
Description: The NUE of wild-type and mutants defective in candidate TFs. Key to the sample ID: Val# indicates the batch the plant was grown.

File Name: Supplementary Data 6
Description: The information of primers used in this paper.

File Name: Supplementary Data 7
Description: Software information for processing RNA-seq libraries

File Name: Supplementary Data 8
Description: The mapping file of Arabidopsis genes and their corresponding maize homologs using Phytozome 10.

File Name: Supplementary Data 9
Description: The feature importance scores of rice gene features in XGBoost fecundity model.
