# Supplementary material for: Evolutionarily informed machine learning enhances the power of predictive gene-to-phenotype relationships
Source: Nat Commun. 2021 Sep 24;12:5627. doi: 10.1038/s41467-021-25893-w (PMC8463701; doi:10.1038/s41467-021-25893-w)
Supplement: Supplementary file 13 — Reporting Summary [file 41467_2021_25893_MOESM13_ESM.pdf]

Corresponding author(s): *Double-blind peer review submissions: write DBPR and your manuscript number here instead of author names.*

Last updated by author(s): *YYYY-MM-DD*

Reporting Summary

Nature Portfolio wishes to improve the reproducibility of the work that we publish. This form provides structure for consistency and transparency in reporting. For further information on Nature Portfolio policies, see our Editorial Policies and the Editorial Policy Checklist.

Statistics

For all statistical analyses, confirm that the following items are present in the figure legend, table legend, main text, or Methods section.

n/a	Confirmed	
☐	☒	The exact sample size (*n*) for each experimental group/condition, given as a discrete number and unit of measurement
☐	☒	A statement on whether measurements were taken from distinct samples or whether the same sample was measured repeatedly
☐	☒	The statistical test(s) used AND whether they are one- or two-sided<br>*Only common tests should be described solely by name; describe more complex techniques in the Methods section.*
☒	☐	A description of all covariates tested
☐	☒	A description of any assumptions or corrections, such as tests of normality and adjustment for multiple comparisons
☐	☒	A full description of the statistical parameters including central tendency (e.g. means) or other basic estimates (e.g. regression coefficient) AND variation (e.g. standard deviation) or associated estimates of uncertainty (e.g. confidence intervals)
☐	☒	For null hypothesis testing, the test statistic (e.g. *F*, *t*, *r*) with confidence intervals, effect sizes, degrees of freedom and *P* value noted<br>*Give P values as exact values whenever suitable.*
☒	☐	For Bayesian analysis, information on the choice of priors and Markov chain Monte Carlo settings
☒	☐	For hierarchical and complex designs, identification of the appropriate level for tests and full reporting of outcomes
☐	☒	Estimates of effect sizes (e.g. Cohen's *d*, Pearson's *r*), indicating how they were calculated

Our web collection on statistics for biologists contains articles on many of the points above.

Software and code

Policy information about availability of computer code

Data collection	The software/parameters for sequencing reads acquisition and processing steps are included in Table S7.
Data analysis	The software/code information is provided in the "Code Availability" section, Table S7 and the Coruzzi lab OSF project page (https://osf.io/avjph/)

For manuscripts utilizing custom algorithms or software that are central to the research but not yet described in published literature, software must be made available to editors and reviewers. We strongly encourage code deposition in a community repository (e.g. GitHub). See the Nature Portfolio guidelines for submitting code & software for further information.

Data

Policy information about availability of data

All manuscripts must include a data availability statement. This statement should provide the following information, where applicable:

- Accession codes, unique identifiers, or web links for publicly available datasets
- A description of any restrictions on data availability
- For clinical datasets or third party data, please ensure that the statement adheres to our policy

Data Availability
The raw and processed data generated in this study including FASTQ, BAM, and read counts have been deposited in the Gene Expression Omnibus (GEO) under accession code GSE152249 [https://www.ncbi.nlm.nih.gov/geo/query/acc.cgi?acc=GSE152249]. Source data are provided with this paper and specified in the legend of each figure.
Code Availability
The code used in this study is available at the Coruzzi lab Open Science Framework (DOI 10.17605/OSF.IO/AVJPH) [https://osf.io/avjph/].

Field-specific reporting

Please select the one below that is the best fit for your research. If you are not sure, read the appropriate sections before making your selection.

- [x] Life sciences
- [ ] Behavioural & social sciences
- [ ] Ecological, evolutionary & environmental sciences

For a reference copy of the document with all sections, see nature.com/documents/nr-reporting-summary-flat.pdf

Life sciences study design

All studies must disclose on these points even when the disclosure is negative.

Sample size	For Arabidopsis lab experiment, the sample size was determined based on a pilot study conducted by Chia-Yi Cheng with the goal to achieve more the 3 data points per biological replicate, which is more than 9 data points per condition per genotype. For maize, each data point is composed of a pool of 5 plants, which was based on the former study by the Moose lab (Lauter et al., 2005).
Data exclusions	No data was excluded from the analysis.
Replication	The Arabidopsis lab experiments were repeated 3 times. The maize field experiments were conducted annually for three consecutive years (2014-2016). All attempts at replication were successful for the experiments listed in this study were successful.
Randomization	Randomization is not relevant to this study because every genotype was provided the same treatment.
Blinding	The investigator was not blinded during data collection and/or analysis. During the analysis (machine learning pipeline), each and every genotype was left out as the test data set in a round robin manner. This approach was meant to demonstrate the result was not biased nor driven by any particular genotype and achieve the same purpose of blinding.

Reporting for specific materials, systems and methods

We require information from authors about some types of materials, experimental systems and methods used in many studies. Here, indicate whether each material, system or method listed is relevant to your study. If you are not sure if a list item applies to your research, read the appropriate section before selecting a response.

Materials & experimental systems

n/a	Involved in the study	
x		Antibodies
x		Eukaryotic cell lines
x		Palaeontology and archaeology
x		Animals and other organisms
x		Human research participants
x		Clinical data
x		Dual use research of concern

Methods

n/a	Involved in the study	
x		ChIP-seq
x		Flow cytometry
x		MRI-based neuroimaging
